# Increased mucosal eosinophils in colonic diverticulosis and diverticular disease

**DOI:** 10.1111/jgh.16278

**Published:** 2023-07-06

**Authors:** Raquel Cameron, Marjorie M. Walker, Michael Jones, Guy D. Eslick, Simon Keely, Peter Pockney, Carolina C. Cosentino, Nicholas J. Talley

**Affiliations:** ^1^ College of Health, Medicine and Wellbeing University of Newcastle Newcastle Australia; ^2^ NHMRC Centre for Research Excellence in Digestive Health New Lambton Heights Australia; ^3^ Hunter Medical Research Institute Newcastle Australia; ^4^ Hunter New England Area Health Service Newcastle Australia; ^5^ Department of Psychology Macquarie University North Ryde Australia

**Keywords:** Anatomical histopathology, Diverticular disease, Diverticulitis, Diverticulosis, Eosinophil, Epidemiology, Gastroenterology, Inflammation

## Abstract

**Aims:**

Eosinophils contribute to tissue homeostasis, damage, and repair. The mucosa of colonic diverticula has not been evaluated for eosinophils by quantitative histology. We aimed to investigate whether mucosal eosinophils and other immune cells are increased in colonic diverticula.

**Methods:**

Hematoxylin and eosin stained sections from colonic surgical resections (*n* = 82) containing diverticula were examined. Eosinophils, neutrophils, and lymphocytes, in five high power fields in the lamina propria were counted at the base, neck, and ostia of the diverticulum and counts compared to non‐diverticula mucosa. The cohort was further subgrouped by elective and emergency surgical indications.

**Results:**

Following an initial review of 10 surgical resections from patients with diverticulosis, a total of 82 patients with colonic resections containing diverticula from the descending colon were evaluated (median age 71.5, 42 M/40F). Eosinophil counts for the entire cohort were increased in the base and neck (median 99 and 42, both *P* = <0.001) compared with the control location (median 16). Eosinophil counts remained significantly increased in the diverticula base (both *P* = <0.001) and neck (*P* = 0.01 and <0.001, respectively) in both elective and emergency cases. Lymphocytes were also significantly increased at the diverticula base compared to controls in both elective and emergency subgroups.

**Conclusion:**

Eosinophils are significantly and most strikingly increased within the diverticulum in resected colonic diverticula. While these observations are novel, the role of eosinophil and chronic inflammation is as yet unclear in the pathophysiology of colonic diverticulosis and diverticular disease.

## Introduction

The pathogenesis of colonic diverticulosis and diverticular disease (DD) remains elusive.^1^ Research to date is limited in identifying the etiology and pathophysiology of diverticula formation although it has been hypothesized that abnormal colonic motility induces exaggerated segmental contractions, resulting in diverticula developing as herniations of the mucosa at points of wall weakness, where vessels penetrate the muscularis,^2^ termed taenia coli,^3^ at the point where the hypertrophic^4^ circular muscle meets the blood vessels.^5^ The formed diverticula are composed of a bulb‐like sac with a thinned layer of muscularis mucosae,^5^ embedded in loose sub‐serosal fibroadipose tissue.^6^ In Western industrialized countries,^2^ diverticulosis is currently the fifth most commonly diagnosed gastrointestinal (GI) disease,^7^ with the sigmoid or rectosigmoid the most frequent site of disease.^4^, ^5^ Risk factors for the development of colonic diverticula include smoking, obesity, and diet.^8^

Colonic diverticulosis^9^ is asymptomatic in the majority of individuals.^4^ In patients who are diagnosed with diverticulosis, only a small minority (4%) develop symptoms.^2^ DD encompasses a disease spectrum that ranges from non‐inflammatory symptomatic uncomplicated diverticular disease (SUDD) to acute, chronic, or complicated diverticulitis^10^ and segmental colitis‐associated diverticulosis (SCAD).^4^

In diverticulosis with or without symptoms, previous studies have reported no evidence of colonic inflammation^10^, ^11^ at microscopy, in contrast to diverticulitis.^5^ However, these studies have been confined to tissue obtained away from the diverticula itself.

A search of the literature that we conducted up to August 2022 using the search terms eosinophil*, histology, diverticul*, count*, and colon* failed to find any papers quantitatively evaluating the presence of eosinophils in the actual colonic diverticula themselves by histopathology in patients with diverticulosis. Therefore, the potential pathophysiological role of eosinophils in colonic diverticula is unknown. Further, eosinophil counts are not routinely evaluated, nor noted in postoperative histopathology reports in our clinical experience. However, we noticed increased eosinophils in our resected cases of diverticulosis.

Therefore, we postulated eosinophils may play a role in the pathogenesis of DD. In this retrospective cohort study, we aimed to investigate eosinophils, neutrophils, and lymphocytes by quantification within the base, neck, and ostia of the diverticulum in patients who had undergone colonic resection surgery for colorectal cancer (CRC) and DD, but away from any macroscopically diseased areas, to determine if these inflammatory cells were increased, compared to non‐diverticula mucosal tissue.

## Methods

### Cohort selection and inclusion and exclusion criteria

Initially, 10 high anterior colonic resections samples (four males, six females) (Table S1) undergoing elective surgery for CRC were selected for a pilot study. Following the positive pilot study showing increased tissue eosinophilia, an additional 72 consecutive patients were added. The total cohort of 82 colonic resection histological samples (42 males, 40 females) (Table 1) comprised patients undergoing resection surgery with in situ diverticula upstream (Table S2).

**Table 1:** Histopathological changes by site. Combined statistics are provided for patients with diverticula at colonic resection (*n* = 82). Statistical counts for eosinophils, and density cell grading scores for neutrophils, lymphocytes, and plasma cells; counted at the base, neck, ostia of the diverticulum, analyzed against control site. Pairwise comparisons with *P*‐value indicated. Subsequent columns are the same but for subtypes: elective (*n* = 37) and emergency (*n* = 45).

Surgical indication	Combined	Elective cohort	Emergency cohort
Covariates	*n* = 82	*n* = 37	*n* = 45
Age (mean [range])	67.96 (23–95)	70.22 (37–91)	66.11 (23–95)
(median)	71.5	72	69
Sex (M/F ratio)	42/40	19/18	23/22
Comorbidities
CRC	19	16	3
IBD	1	0	1
Other colonic disease	7	2	5
Obesity	9	3	6
Type 2 diabetes	13	9	4
Asthma	13	7	6
Other atopy	3	2	1
History of smoking	35	18	17
Medication/therapy
Neo/adjunct therapy	7	6	1
Antibiotics pre‐OT	23	4	19
PPIs	24	14	10
NSAIDs	22	14	8
Cell count analysis
Eosinophils
Base			
Median Mean Standard deviation Standard error of mean	98 102 58 6.5	122 122 65 11	92 86 47 7.0
Neck			
Median Mean Standard deviation Standard error of mean	42 45 33 3.7	45 51 36 6.1	31 40 30 4.5
Ostia			
Median Mean Standard deviation Standard error of mean	21 30 23 2.7	25 34 25 4.3	19 26 21 3.3
Control			
Median Mean Standard deviation Standard error of mean	16 22 18 2.0	18 26 19 3.2	14 19 16 2.4
*P* value: Base	<0.001***	<0.001***	<0.001***
Neck	<0.001***	0.005**	<0.001***
Ostia	0.30	0.66	0.42
Neutrophils
Base			
Median Mean Standard deviation Standard error of mean	0.00 1.2 2.8 0.31	0.0 1.1 3.1 0.51	0.00 1.4 2.5 0.37
Neck			
Median Mean Standard deviation Standard error of mean	0.00 0.49 2.3 0.26	0.0 0.32 1.0 0.17	0.0 0.63 3.0 0.37
Ostia			
Median Mean Standard deviation Standard error of mean	0.00 0.49 2.1 0.25	0.0 0.19 0.59 0.10	0.0 0.73 2.8 0.43
Control			
Median Mean Standard deviation Standard error of mean	0.00 0.20 0.96 0.11	0.0 0.03 0.17 0.03	0.0 0.34 1.3 0.19
*P* value: Base	0.003**	0.02*	0.07
Neck	0.69	0.81	0.88
Ostia	0.61	0.96	0.61
Lymphocytes
Base			
Median Mean Standard deviation Standard error of mean	110 190 178 20	110 185 171 28	110 194 185 28
Neck			
Median Mean Standard deviation Standard error of mean	56 70 66 7.5	56 59 32 5.4	56 78 83 13
Ostia			
Median Mean Standard deviation Standard error of mean	43 49 29 3.4	45 54 35 6.2	42 45 24 3.8
Control			
Median Mean Standard deviation Standard error of mean	38 40 19 2.2	39 43 23 3.8	37 37 16 2.4
*P* value: Base	<0.001***	<0.001***	<0.001***
Neck	0.13	0.80	0.15
Ostia	0.90	0.94	0.97

The patients were selected from a larger cohort (*n* = 388) (Fig. S1) obtained by running a query of the histopathology database at Hunter New England Health Pathology (HNEAHP), Newcastle, Australia, specifying the years 2017–2019, for all patients using key search words of “diverticulosis,” “diverticulitis,” or “diverticular disease.” The HNEHP database was further queried, enabling the exclusion of records that contained diverticula not located in the colon and patient duplicates (*n* = 31 removed). We also excluded records that did not include specimens from a colonic surgical resection (*n* = 129 removed). The remaining cohort (*n* = 228) was checked against HNEHP histopathology reports to ensure that postsurgical histology reports identified that all colonic diverticula examined were upstream of any mass, obstruction, or cancer (*n* = 45 removed). The last step that determined inclusion in the study involved screening hematoxylin and eosin (H&E) tissue sections for well‐orientated sections, sufficient adjacent non‐diseased mucosa, thus making them suitable for histological assessment and cell counting (*n* = 101 removed). Eighty two patients met the a priori study criteria.

A second consecutive control cohort (*n* = 10) was selected using identical selection criteria with the exception that this surgical group contained no observed diverticula at histopathology.

### Ethics approval

Approval for ethical exemption as an assessment of negligible risk research activity was sought from the Hunter New England Local Health District's Human Research Ethics Committee. The exemption was authorized on March 30, 2020. Approval identification: AU202003‐10.

### Covariate data

For all participants, covariate data were collated from pre‐admission surgical request forms (Table 1). For other potentially relevant comorbidities, the patient's medical records were searched for data that indicated a previous history of IBD, other colonic diseases, obesity, type 2 diabetes mellitus, asthma or other respiratory diseases, other atopic diseases, and smoking history.

Patients' preoperative use of neo/adjunctive therapy, and antibiotics, as well as routine administration of proton pump inhibitors (PPIs) and nonsteroidal anti‐inflammatory drugs (NSAIDs), were also recorded from preoperative administrational records.

### Histopathology screening

**Figure 1:**
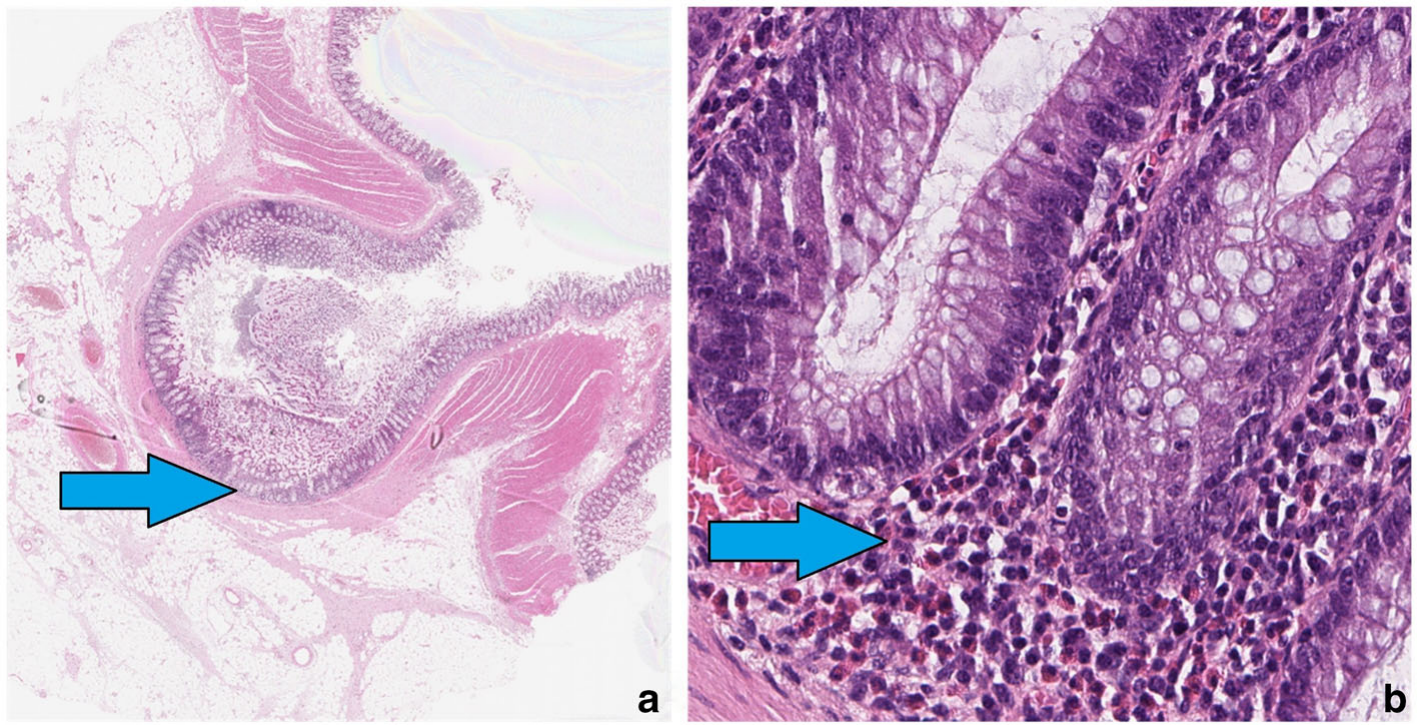
Histopathology: colonic diverticulum with eosinophils indicated by blue arrows. Diverticulum observed digitally. H&E resection tissue from the sigmoid colon. (a) 0.5× magnification of diverticulum diagnosed as diverticulosis. (b) 40× magnification showing eosinophils in the base of lamina propria at the base of the diverticulum bulb.

**Figure 2:**
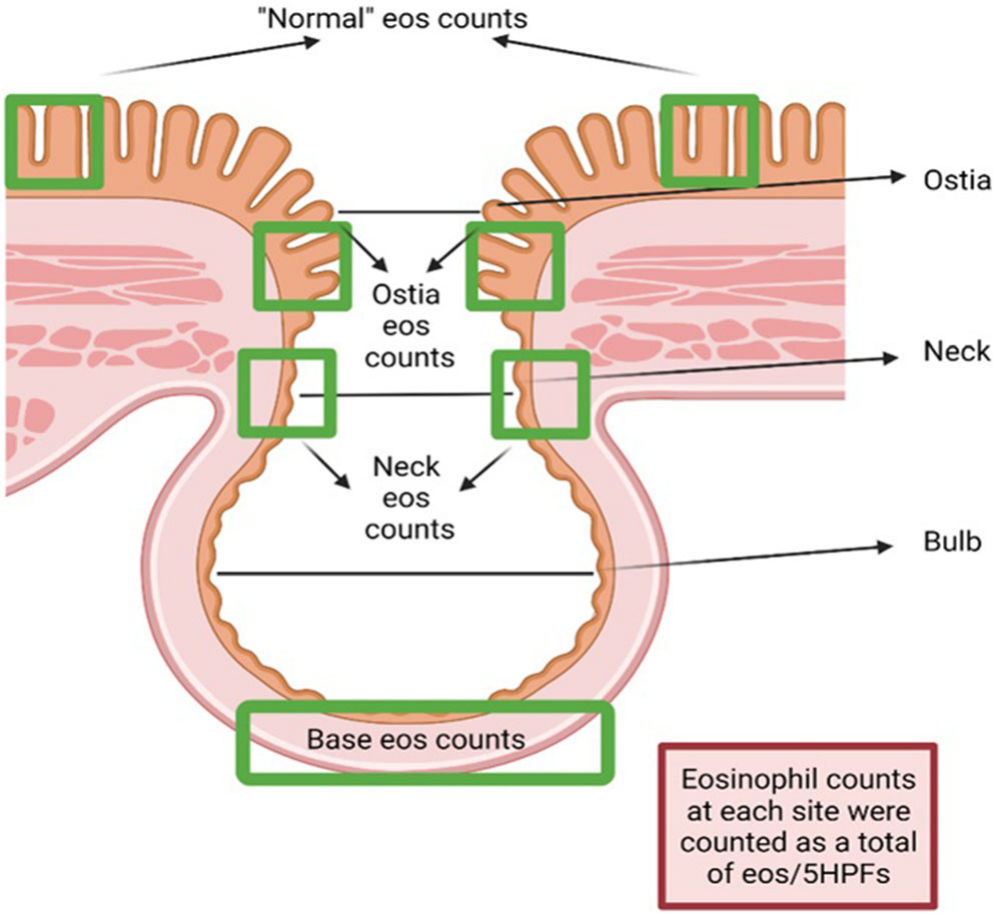
Graphical image of a diverticulum showing regions at which cell counts were performed. 5HPF (high power fields) eosinophil (eos) counts were obtained at the base, neck, ostia, and control (non‐diverticula mucosal tissue). Image by BioRENDER.com.

Hematoxylin and eosin stained histological sections obtained from the Pathology North Laboratory at the John Hunter Hospital (JHH), Newcastle, Australia, were digitally scanned on an Aperio AT2 Digital Pathology Scanner (Leica Biosystems). The cell counts were performed using the software package Aperio ImageScope (v12.4.3.5008, Leica Biosystems) by two independent observers (RC/MMW) who were blinded to the patient's status. At 40× resolution in five high power field (HPF) equivalents, each within a grid of 100 × 100 μm, eosinophils, neutrophils, and lymphocytes, at the base, neck, and ostia of the diverticulum, were counted (Fig. 1). Similarly, sections from the same patient were assessed, which were defined as “normal,” or un‐inflamed, non‐diverticula tissue (Fig. 2 and Table S2) and termed as “controls” for this study. HPFs were selected by scanning the mucosa adjacent to the diverticulum and counting the five areas consisting of the highest density by eye or the single observed area of highest density and the surrounding HPFs. Normal eosinophil counts were defined a priori as <25 in any single HPF as per the diagnostic criteria for eosinophilic colitis and colonic eosinophilia.^12^

The second control group of non‐diverticular resections were screened as per the diverticula‐inclusive cohort. In the absence of a diverticulum, the lamina propria was observed at 20× resolution for areas of the highest eosinophil, neutrophil, and lymphocyte cell densities. Counts were performed as per diverticular samples.

### Statistical analysis

Statistics were carried out using software packages STATA (v17, StataCorp LLC) and GraphPad Prism (v.9.3.1, GraphPad Software). Chi‐square testing for categorical measures was undertaken for comorbidities and presurgical treatments. Linear mixed models were used for diverticular histopathology results accounting for within‐subject correlation due to repeated measurement across diverticulum sites. An overall (omnibus) test of equality across sites was undertaken followed by contrasts of each site with the control site (analysis of variance mixed‐affect analysis with Dunnett's multiple comparison test correction). The two control cohorts (tissue from patients who do not have colonic diverticula and tissue from patients with colonic diverticula taken from the non‐diverticula region) were compared by two‐tailed Mann–Whitney test of comparison to show that there was no significant difference between the two control groups.

## Results

Histopathology was independently carried out by two observers who were blinded to each other's counts, with eosinophils, neutrophils, and lymphocytes counted independently in the same HPFs on digital images, achieving good agreement. Reliability of interobserver agreement was assessed by Wilcoxon two‐tailed nonparametric paired *t*‐test, which showed that the matched pairs had very high concordance (one‐tailed: *P* = <0.001***). Spearman correlation coefficient ((r): 0.99) showed effective and significant pairing.

In the pilot study (*n* = 10; median age of 70), we observed a clear and significant increase in eosinophil counts in the base of the diverticulum bulb (median 135.5; *P* = <0.001) compared to control counts (Table S1). The counts at the diverticulum neck and ostia were not significantly different from the controls (Fig. S2).

Lymphocytes were also significantly increased within the bulb base (median 115 115, *P* = <0.001) (Fig. S3).

**Figure 3:**
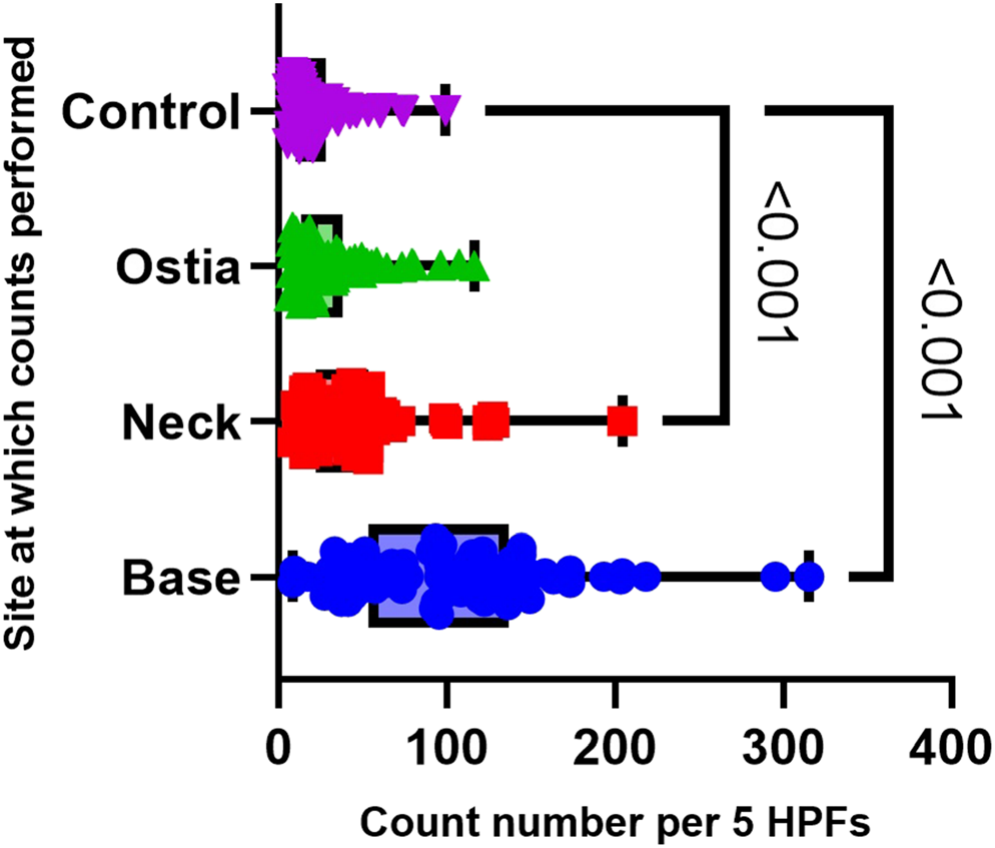
Eosinophil counts as a whole cohort (*n* = 82). Box and violin plot showing the count sites: base, neck, and ostia, against the control site. Count numbers are indicated as numbers per 5HPFs. Pairwise comparisons with *P*‐value indicated. Median with 95% CI.

In the total cohort (*n* = 82; median age 71.5 [23–95]), eosinophil counts were strikingly increased in the base and neck (median 98 and 42, both *P* = <0.001) when compared with the control counts (median 16) (Fig. 3). Counts at the ostia were not statistically significant.

**Figure 4:**
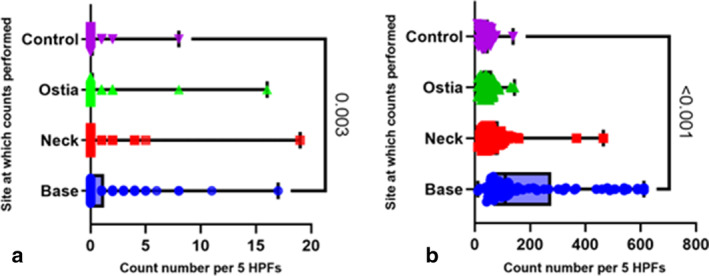
Neutrophil and lymphocyte cell counts in the study (*n* = 82). Box and violin plot showing (a) neutrophil and (b) lymphocyte counts performed at the diverticulum site: base, neck, and ostia against the control site. Count numbers are indicated as number per 5HPFs. Pairwise comparisons with *P*‐value indicated. Median with 95% CI.

Lymphocytes were significantly increased in the base of the diverticulum versus controls (medians110 and 3838, respectively) (*P* = <0.001) (Fig. 4). None of the histology showed evidence of inter‐diverticular colitis.

**Figure 5:**
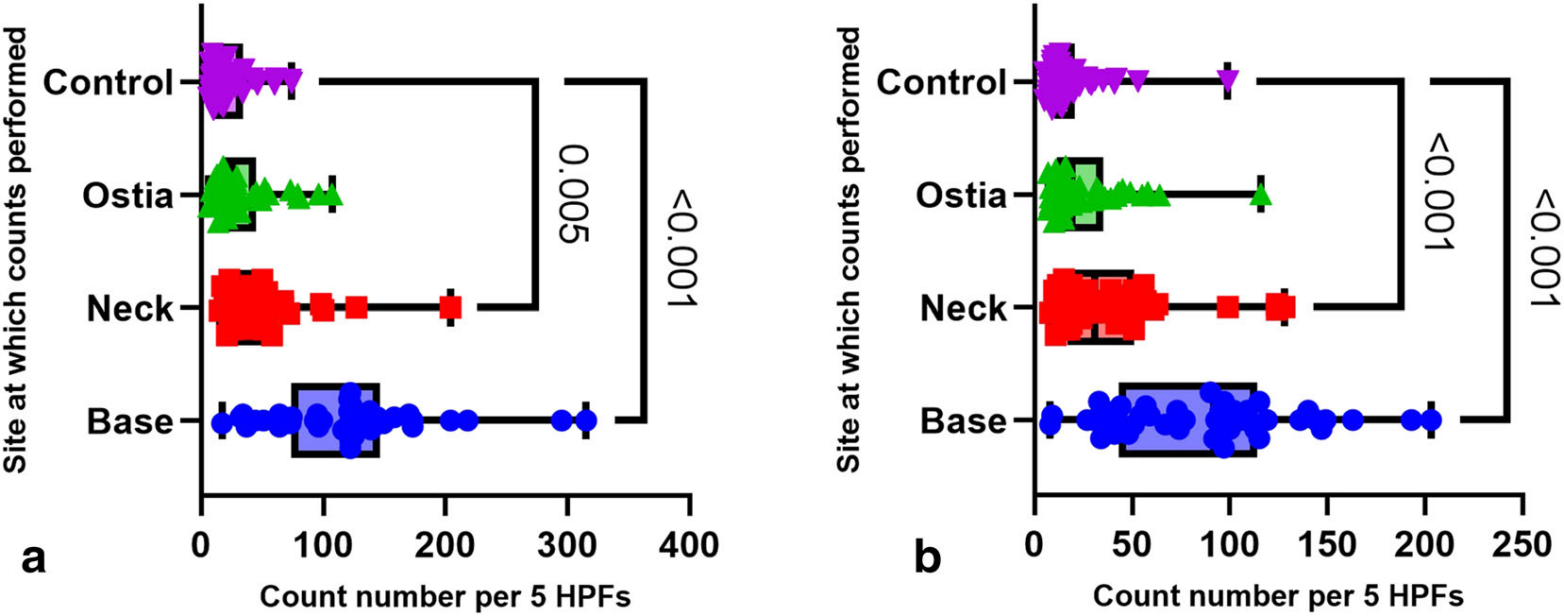
Eosinophil counts for subgroups elective and emergency DD. Box and violin plot showing cell counts performed per site: base, neck, ostia against control for patients with diverticula at colonic resection for the subgroups (a) elective (*n* = 37) and (b) emergency (*n* = 45). Count numbers are indicated as numbers per 5HPFs. Pairwise comparisons with *P*‐value indicated. Median with 95% CI.

The entire cohort was further subgrouped into patients who underwent elective (*n* = 37, median age 72 [37–91], 19 male), and emergency resection surgery (*n* = 45, median age 66.11 [23–95], 23 male). Both elective and emergency subgroups had significantly increased eosinophils at the base (median 122 and 92, both *P* = 0.001), and neck (median 45 and 31, *P* = 0.005 and 0.001, respectively) of the diverticulum. There was no significant increase in eosinophil counts at the ostia by subgroup (Fig. 5).

To test the age association of colonic diverticulosis and increased eosinophil cell numbers, a comprehensive comparison of age ranges (Table S4) for the entire cohort and subgroups was analyzed by eosinophil counts. All age ranges had statistically significant eosinophil counts at the base of the diverticulum.

**Figure 6:**
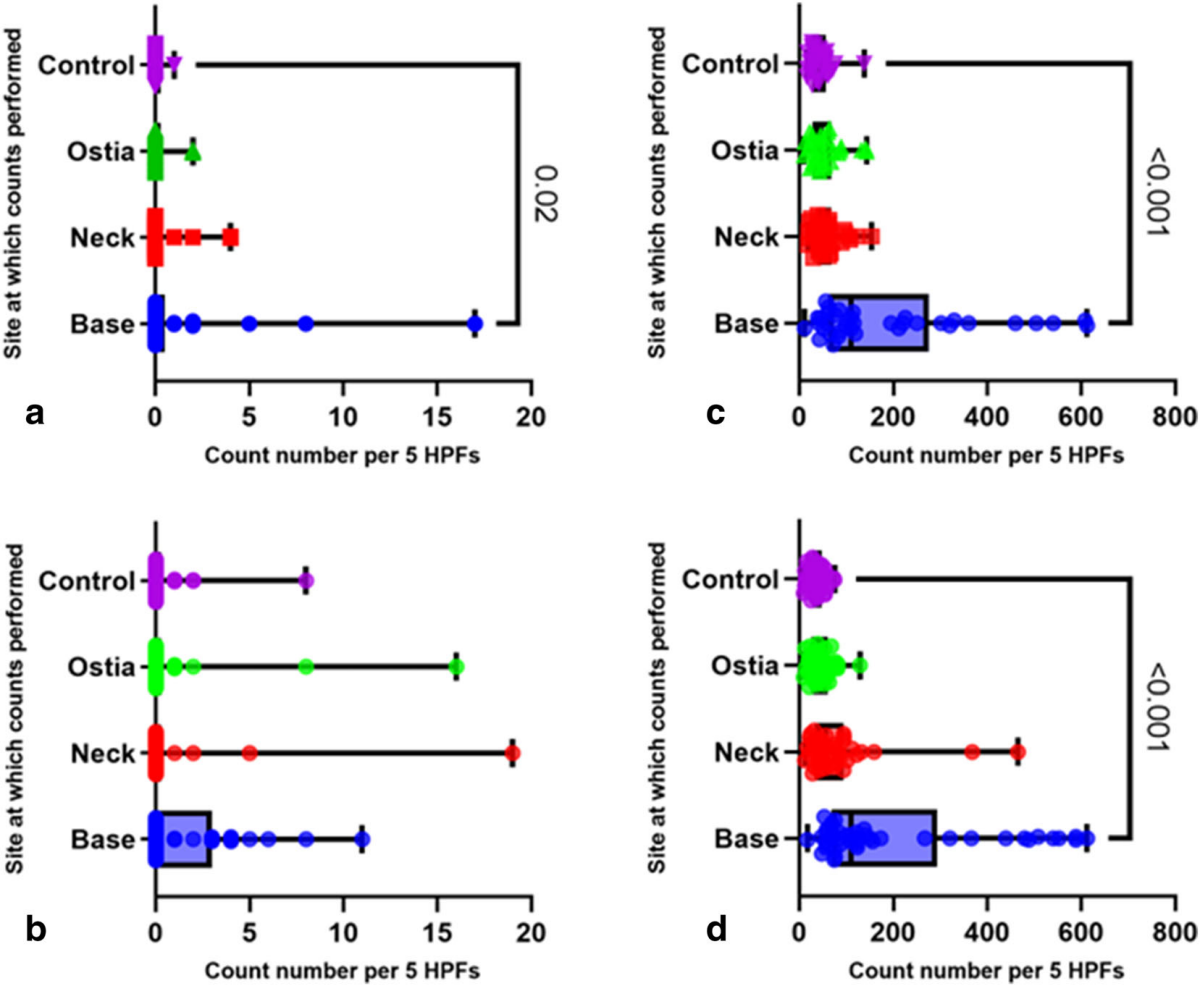
Neutrophil, and lymphocyte cell counts in subgroups elective (*n* = 37) and emergency (*n* = 45). Box and violin plot showing (a,b) for neutrophil cell counts and (c,d) for lymphocyte cell counts, for sites: base, neck, and ostia, against control site. Count numbers are indicated as numbers per 5HPFs. Top row (left to right) shows elective subgroup, and bottom row (left to right) shows emergency subgroup. Pairwise comparisons with *P*‐value indicated. Median with 95% CI.

For both subgroups, lymphocyte counts were significantly increased in the base (both *P* = <0.001) of the diverticulum compared to controls. Neutrophil cell densities were only significantly increased in the base (*P* = 0.202) of elective patients (Fig. 6).

To test the validity of the control data, a second non‐diverticula control cohort was assessed for colonic eosinophil, neutrophil, and lymphocyte cell counts. There was no significant difference between the control groups (*P* = 0.43, >0.99, and 0.95, respectively) (Table S3).

Demographic and comorbidity data were similar among subgroups (Table S5). There was a higher rate of CRC cases in the elective subgroup (OR 11[95% CI 3.0–36]; *P* = 0.0001). Treatments undertaken before surgery were also at a higher rate in the elective subgroup for neoadjuvant therapy (OR 8.5[95% CI 1.2–100]; *P* = 0.04) and NSAIDs (OR 2.8[95% CI 1.0–8.1]; *P* = 0.049), while the emergency subgroup was more frequently prescribed antibiotics presurgery (OR 0.17[95% CI 0.057–0.55]; *P* = 0.0026). PPI use between the two subgroups was not significantly different (OR 2.1 [1.0–8.1]; *P* = 0.15).

## Discussion

In patients undergoing colonic resection, eosinophil numbers at the base and neck of the diverticulum were strikingly increased compared with non‐diverticula mucosal tissue in the same patients. Eosinophils were the dominant inflammatory infiltrate in these diverticulum regions, defined overall by >25 eosinophils per HPF, counts consistent with “eosinophilic colitis” and “colonic eosinophilia.”^13^, ^14^

In this study, a pattern of chronic inflammation characterized by a statistically significant but modest increase in lymphocytes,^15^ was also observed at the diverticulum base and neck. This suggests a unique inflammatory pattern not previously reported, which we suggest together is consistent with the term “microscopic colitis not otherwise specified.”^5^, ^15^ It cannot be ascertained from this data if this inflammatory pattern occurs before the formation of the diverticulum or is secondary to the presence of the diverticulum. It is interesting to note that the increase of eosinophils and other inflammatory cells in the bulb base and neck of the diverticulum was statistically significant regardless of elective or emergency surgical indication. There was not an association of increasing inflammation with increasing age with analysis of age groups indicating that even from a younger age, there is an association of colonic diverticula and increased eosinophils.^2^ Studies on the surgical stress response from colorectal resection^16^, ^17^ have indicated that circulating inflammatory cell levels are only elevated in the postsurgical recovery period and are within normal parameters preoperatively. Further, each patient acted as their internal control in this study. Therefore, we can confidently rule out surgical stress as a major cause of the increased levels of eosinophils and other immune cells in the base of the diverticula.

Previous research on eosinophils has identified different roles and functions for resident (rEos) and induced (iEos) tissue eosinophils.^18^ Intestinal rEos can be distinguished from iEos by their surface receptor expression (intermediate Siglec‐8 levels, a high cluster of differentiation (CD)62L levels, low CD101 levels and not IL‐5 dependent).^19^ At the steady state, intestinal rEos are not allergen‐induced and exhibit an immunosuppressive role, while iEos are antigen‐induced (with high Siglec‐8 and CD101 levels, low CD62L levels, and are IL‐5 dependent), which is proinflammatory.^20^

Eosinophils in homeostasis, as postulated in the Local Immunity And/or Remodeling/Repair (*LIAR)* hypothesis,^21^ are intrinsically homeostatic cells undertaking functional roles in local immunity through inflammation control, tissue remodeling, and epithelial barrier maintenance and repair.^22^ Further to this, they are not exclusively linked with innate host defense mechanisms or immune dysregulation but can accumulate to ensure tissue homeostasis is maintained in both healthy and pathological states. This occurs after tissue damage has been induced by pathogens, toxins, or post‐cell death. In homeostasis, 5–25% of circulating eosinophils will migrate to the GI tract.^23^ Eosinophils are most commonly resident in the lamina propria,^24^ with numbers increasing distally (stomach to caecum)^22^ and then decreasing along the colon from the caecum to the rectosigmoid. In secondary eosinophilic disorders such as IBD,^25^ eosinophils can protect the mucosal barrier but also elicit structural alterations^18^ being both anti‐inflammatory by linking the innate and adaptive immunity^22^ and pro‐inflammatory mediating tissue damage through degranulation and the release of toxic granule proteins.^21^ Therefore, following damage caused to the mucosa and muscularis layers when the diverticulum herniates through the colon wall, it could be hypothesized that eosinophils are stimulated, perhaps secondary to tissue hypoxia^26^ in response to the formation of the diverticulum itself. Eosinophils function as proangiogenic cells producing transforming growth factor (TGF)‐β, fibroblast growth factor, and vascular endothelial growth factor (VEGF)^26^, ^27^ and may enter the spaces within the colonic wall to promote the healing of the structural damage caused by the colonic wall herniation when the circular muscle becomes thickened with elastin deposition.^6^, ^28^ Based on this study, it is now our hypothesis that increased colonic diverticula eosinophils may be rEos recruited by structural changes elicited during diverticula formation. The characterization of eosinophil subtypes in the colonic diverticula is one of the next steps required to understand their role.

We were not able to assess risk factors for the eosinophilic inflammation as strikingly all cases had increased eosinophils in the diverticula bases. Smoking history was reported by 41% of the combined cohort but was similar in emergency and elective cases (OR 1.6(95% CI 0.63–3.6); *P* = 0.37). Previous studies have reported that the nicotine in cigarette smoke inhibits the synthesis of proinflammatory cytokines such as IL‐1 and tumor necrosis factor (TNF) and may alter the microbiome.^29^, ^30^ Another study has demonstrated that smoking increases nitric oxide.^31^ The link between the microbiome and nitric oxide (NO) in colonic diverticula has been researched by Turco *et al*.^32^ in SUDD patients, where it was observed bacterial dysbiosis increases the expression of NO. However, it appears that smoking status did not confound the observation of inflammatory cell densities in the diverticula compared to the rest of the colon in this cohort.

Presurgery, the elective subgroup was more likely to be taking PPIs or NSAIDs. Previous studies have indicated that the use of NSAIDs in patients with colonic diverticula increases the risk of diverticulitis complications and bleeding compared to controls,^33^ but this seems unlikely to explain our results. PPI use in colonic DD has previously been assessed,^34^, ^35^ and it has been reported it can induce dysbiosis in the colon through the suppression of gastric acid leading to bacterial colonization and overgrowth. However, so far, these same studies have not attributed the use of PPIs to the risk of developing diverticulitis.

The limitations of this study need to be considered. The sample size was reasonable but despite a rigorous selection process, the cases may not fully represent the population of DD patients. To obtain diverticular full‐thickness samples for research, surgical intervention is required. Therefore, capturing a true representation of the community with diverticulosis is unachievable as biopsies cannot be taken at colonoscopy from the base of the diverticulum. Another limitation is the potential for contamination of the diverticulum by surgical indication. We did try to control for this by the exclusion of downstream diverticula. DD includes SUDD and segmental colitis‐associated diverticulitis (SCAD). We were unable to determine if any of our cases had SUDD. None of the histology showed evidence of inter‐diverticular colitis ruling out any SCAD patients in this cohort meeting the definition.

A main strength and novelty of the study was the inclusion of two control groups, the first being normal tissue obtained away from the diverticula and the second recruiting a control cohort. Thus, we were able to confirm that the results obtained away from the diverticula in cases represented a comparable control group for the assessment of eosinophil numbers in diverticulum tissue regions.

In conclusion, we observed that eosinophils are increased in the base and neck of the colonic diverticula. The role of increased eosinophils in the etiology of diverticular formation now needs further investigation. We postulate that eosinophils may play a key role in the regulation of colonic diverticula mucosal homeostasis. The current study does not provide answers as to whether eosinophils are involved in the pathophysiology of the diverticulum formation or disease progression, but it does provide new clues that may help further address the underlying etiopathogenesis of the disease.

## Transparency

The lead author affirms that this manuscript is an honest, accurate, and transparent account of the study being reported; that no important aspects of the study have been omitted; and that any discrepancies from the study as planned (and, if relevant, registered) have been explained.

## Supporting information


**Figure S1.** Flow diagram of cohort selection process. HNEHP: Hunter New England Health Pathology; H&E: haematoxylin and eosin.
**Figure S2.** Eosinophil cell counts in diverticulosis (n = 10). Counted per 5 HPFs, counted at the base, neck, ostia compared to the control site, in the pilot study. Welsh test results graphed on Box and violin plot showing site of counts performed against no of cells counted per site: base, neck, ostia against control. Pairwise comparisons are shown by plot lines with P‐value indicated. Median with 95% CI.
**Figure S3.** Neutrophil (A) and lymphocyte (B) cell counts (n = 10). Counted per 5 HPFs, counted at the base, neck, ostia compared to the control site, in the pilot study. Welsh test results graphed on Box and violin plot showing site of counts performed against no of cells counted per site: base, neck, ostia against control. Pairwise comparisons are shown by plot lines with P‐value indicated. Median with 95% CI.
**Table S1.** Pilot study cohort (n = 10). Data recorded for age (mean, range, and median for overall cohort; and separately the median for males/females). Also, male, and female sex ratios. Secondly, the table provides the total median value and p‐value data for cell count analysis for eosinophils, neutrophils, lymphocytes, and plasma cells at the base, neck, ostia regions of the diverticulum against the control sample obtained within the colonic lumen. *‐**** indicates the level of significance.
**Table S2.** Surgical and histopathology data obtained from hospital dataset. Patients (n = 85) data were retrieved from the hospital and hospital‐based pathology service: surgical groups (elective or emergency admission), indication for surgery noted in hospital records, and reporting of inflammatory (or words describing inflammatory cells, and their location within the diverticulum, and adjacent (within the control region of luminal colonic mucosa: i.e., non‐diverticular region).
**Table S3.** Cell count differences between control cohorts.
**Table S4.** Eosinophil counts by age range. Mean (standard deviation) and p‐value. * Statistically significant (compared to normal).
**Table S5.** Comorbidity statistics between subgroups of elective and emergency subgroup cohorts.
