# Supplementary material for: Increased mucosal eosinophils in colonic diverticulosis and diverticular disease
Source: J Gastroenterol Hepatol. 2023 Jul 6;38(8):1355–64. doi: 10.1111/jgh.16278 (PMC10946982; doi:10.1111/jgh.16278)
Supplement: Supplementary file 1 — Figure S1. Flow diagram of cohort selection process. HNEHP: Hunter New England Health Pathology; H&E: haematoxylin and eosin. Figure S2. Eosinophil cell counts in diverticulosis (n = 10). Counted per 5 HPFs, counted at the base, neck, ostia compared to the control site, in the pilot study. Welsh test results graphed on Box and violin plot showing site of counts performed against no of cells counted per site: base, neck, ostia against control. Pairwise comparisons are shown by plot lines with P‐value indicated. Median with 95% CI. Figure S3. Neutrophil (A) and lymphocyte (B) cell counts (n = 10). Counted per 5 HPFs, counted at the base, neck, ostia compared to the control site, in the pilot study. Welsh test results graphed on Box and violin plot showing site of counts performed against no of cells counted per site: base, neck, ostia against control. Pairwise comparisons are shown by plot lines with P‐value indicated. Median with 95% CI. Table S1. Pilot study cohort (n = 10). Data recorded for age (mean, range, and median for overall cohort; and separately the median for males/females). Also, male, and female sex ratios. Secondly, the table provides the total median value and p‐value data for cell count analysis for eosinophils, neutrophils, lymphocytes, and plasma cells at the base, neck, ostia regions of the diverticulum against the control sample obtained within the colonic lumen. *‐**** indicates the level of significance. Table S2. Surgical and histopathology data obtained from hospital dataset. Patients (n = 85) data were retrieved from the hospital and hospital‐based pathology service: surgical groups (elective or emergency admission), indication for surgery noted in hospital records, and reporting of inflammatory (or words describing inflammatory cells, and their location within the diverticulum, and adjacent (within the control region of luminal colonic mucosa: i.e., non‐diverticular region). Table S3. Cell count differences between control cohorts. Table S4 [file JGH-38-1355-s001.docx]

**Supplementary figures:**

**Figure S1**. **Flow diagram of cohort selection process.**
HNEHP: Hunter New England Health Pathology; H&E: haematoxylin and eosin

**Figure S2.** **Eosinophil cell counts in diverticulosis (n=10).** Counted per 5 HPFs, counted at the base, neck, ostia compared to the control site, in the pilot study. Welsh test results graphed on Box and violin plot showing site of counts performed against no of cells counted per site: base, neck, ostia against control. Pairwise comparisons are shown by plot lines with P-value indicated. Median with 95% CI.


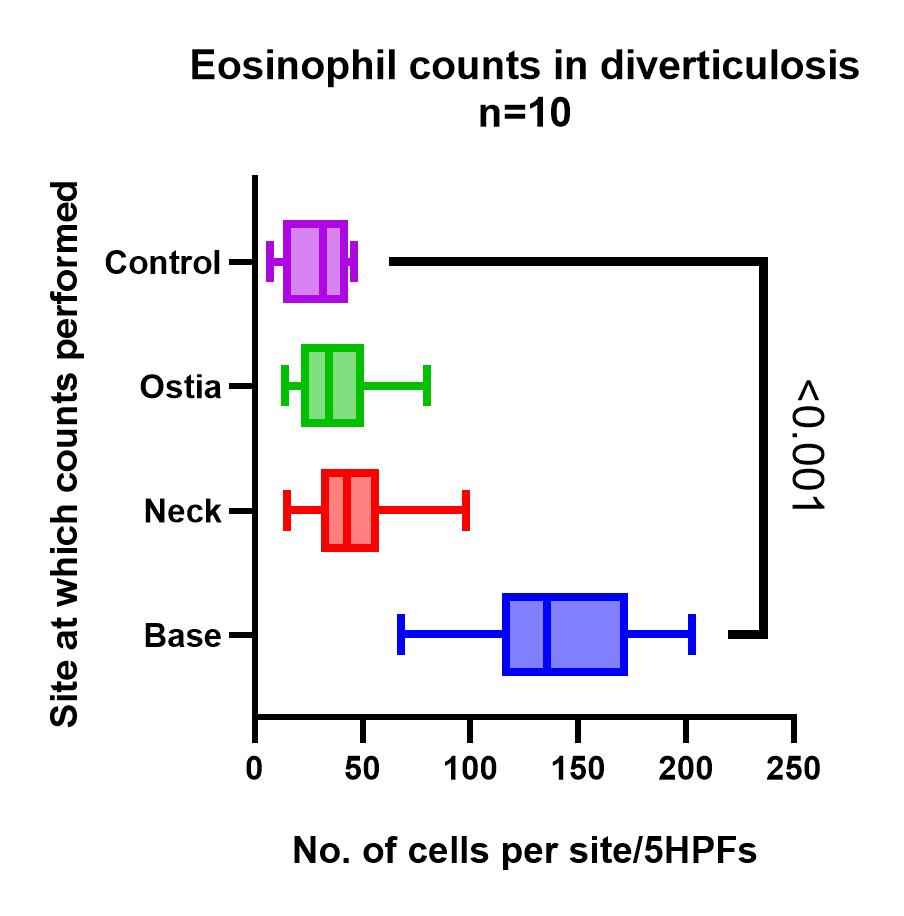


**Figure S3.** **Neutrophil (A) and lymphocyte (B) cell counts (n=10**). Counted per 5 HPFs, counted at the base, neck, ostia compared to the control site, in the pilot study. Welsh test results graphed on Box and violin plot showing site of counts performed against no of cells counted per site: base, neck, ostia against control. Pairwise comparisons are shown by plot lines with P-value indicated. Median with 95% CI.


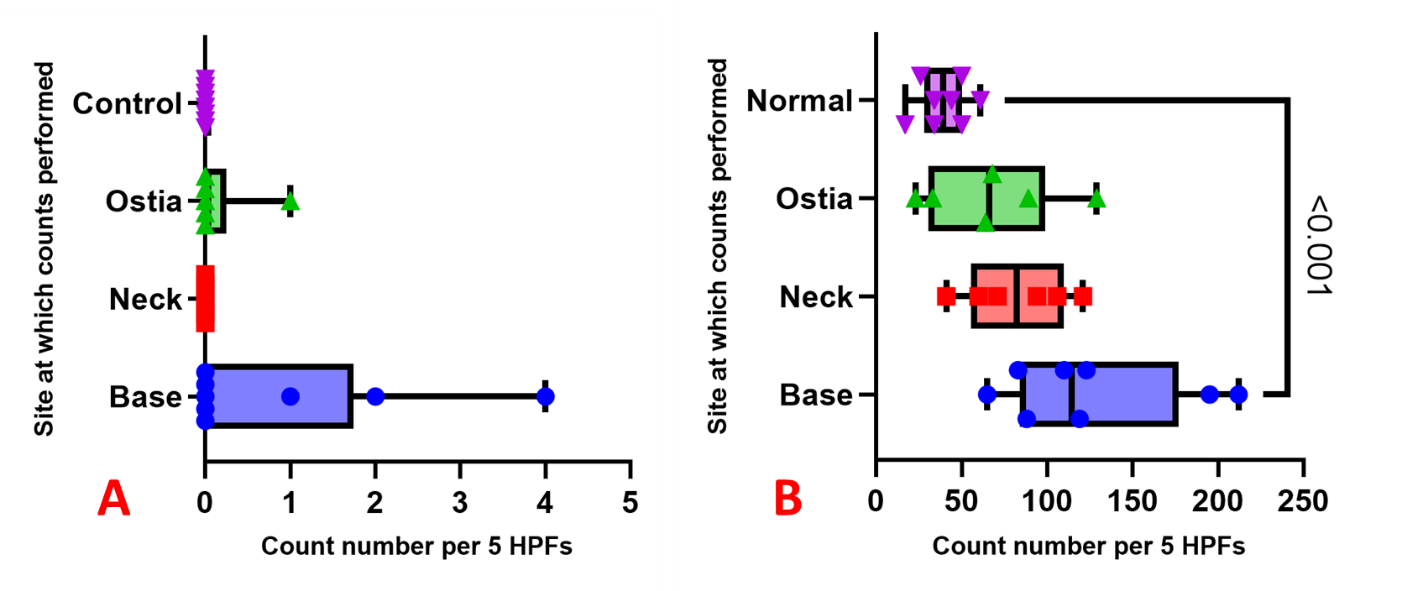


**Table S1.** **Pilot study cohort (n=10).** Data recorded for age (mean, range, and median for overall cohort; and separately the median for males/females). Also, male, and female sex ratios. Secondly, the table provides the total median value and p-value data for cell count analysis for eosinophils, neutrophils, lymphocytes, and plasma cells at the base, neck, ostia regions of the diverticulum against the control sample obtained within the colonic lumen. *-**** indicates the level of significance.

**Supplementary tables:**

| **COVARIATES (N=10)** | **MALES** | | **FEMALES** | | **OVERALL** | |
| --- | --- | --- | --- | --- | --- | --- |
| Age (mean (range)) |  | |  | | 72 (56-88) | |
| (median) | 70 | | 72 | | 70 | |
| Sex (M/F ratio) | 40 | | 60 | |  | |
|  |  | |  | |  | |
| **CELL COUNT ANALYSIS** | **BASE** | **NECK** | | **OSTIA** | | **CONTROL** |
| **EOSINOPHILS (cells/5HPFs)** |  | | | | | |
| **Total Median value p-value** | 135.5  <0.001**** | 43.0  0.27 | | 34.5  0.70 | | 31.5  Reference |
| **NEUTROPHILS (cells/5HPFs)** |  | | | | | |
| **Total Median value**  **p-value** | 0  0.09 | 0  >0.99 | | 0  0.97 | | 0  Reference |
| **LYMPHOCYTES (cells/5HPFs)** |  | | | | | |
| **Total Median value**  **p-value** | 115  <0.001**** | 83  0.10 | | 66  0.36 | | 39  Reference |

**Table S2. Surgical and histopathology data obtained from hospital dataset.** Patients (n=85) data were retrieved from the hospital and hospital-based pathology service: surgical groups (elective or emergency admission), indication for surgery noted in hospital records, and reporting of inflammatory (or words describing inflammatory cells, and their location within the diverticulum, and adjacent (within the control region of luminal colonic mucosa: i.e., non-diverticular region).

| **PATIENT NO.** | **SURGICAL GROUP** | **INDICATION** | **INFLAMMATION WITHIN DIVERTICULUM** | **INFLAMMATION ADJACENT** |
| --- | --- | --- | --- | --- |
| 1 | Elective | Diverticular disease | No | No |
| 2 | Elective | Adenoma rectosigmoid | No | No |
| 3 | Elective | Diverticular disease | No | No |
| 4 | Elective | Colovesical fistula  Diverticular disease | No | No |
| 5 | Elective | Diverticular disease | No | No |
| 6 | Elective | Diverticular disease | Yes: active diverticulitis | No |
| 7 | Elective | Diverticular disease | Yes: active diverticulitis | No |
| 8 | Elective | Colovesical fistula  Diverticular disease | Yes: one diverticulum exhibits inflammation | No |
| 9 | Elective | Adenocarcinoma cecum | Yes: confirms diverticulitis. Elsewhere, diverticular disease without inflammation | No |
| 10 | Elective | Rectal carcinoma | No | No |
| 11 | Elective | Rectal carcinoma | No | No |
| 12 | Elective | Rectal carcinoma | No | No |
| 13 | Elective | Rectal carcinoma | No | No |
| 14 | Elective | Sigmoid carcinoma | No | No |
| 15 | Elective | Rectal carcinoma | No | No |
| 16 | Elective | Sigmoid carcinoma | No | No |
| 17 | Elective | Rectal carcinoma | No | No |
| 18 | Elective | Sigmoid carcinoma | No | No |
| 19 | Elective | Rectal carcinoma | No | No |
| 20 | Elective | Rectal carcinoma | No | No |
| 21 | Elective | Rectal adenoma | No | No |
| 22 | Elective | Rectal prolapse | Yes: there is focal acute inflammation | No |
| 23 | Elective | Diverticular fistula | Yes: foreign body-giant cell reaction to refractile material (faeces) | No |
| 24 | Elective | Sigmoid diverticulitis | No | No |
| 25 | Elective | Colo vesical fistula  Diverticular disease | No | No |
| 26 | Elective | Sigmoid carcinoma | Yes: areas of acute diverticulitis | No |
| 27 | Elective | Transverse colon carcinoma | No | No |
| 28 | Elective | Diverticular stricture sigmoid | No | No |
| 29 | Elective | GIST | No | No |
| 30 | Elective | Ovarian cancer - Mullerian tumour | No | No |
| 31 | Elective | Recurrent diverticulitis | No | No |
| 32 | Elective | Recurrent diverticulitis sigmoid | Yes: two diverticula show foreign body giant cell reaction, otherwise unremarkable. | No |
| 33 | Elective | Recurrent diverticulitis | No | No |
| 34 | Elective | Recurrent diverticulitis | No | No |
| 35 | Elective | Perforated diverticulitis,  multiple abscess drainage | Yes: neutrophils and foreign body type giant cell reaction | Yes: (block 6 only). This block not used in this study. Block 2 used. |
| 36 | Elective | Colo vaginal fistula  Diverticular disease | No | No |
| 37 | Elective | Recurrent diverticulitis | No | No |
| 38 | Emergency | Perforated diverticular disease | Yes: acute | No |
| 39 | Emergency | Perforated diverticular disease | Yes: active acute diverticulitis, others unremarkable | Yes: (blocks 1-3) No: (blocks 4-5)  Block 4 used in this study. |
| 40 | Emergency | Perforation during elective colonoscopy | No | No |
| 41 | Emergency | Perforated diverticular disease | No | No |
| 42 | Emergency | Perforated diverticular disease | Yes: one diverticulum shows inflammation | Yes |
| 43 | Emergency | Perforated diverticular disease | Yes: background active diverticular disease | No |
| 44 | Emergency | Perforated diverticular disease | Yes: diverticular disease with one inflamed diverticulum | Yes: (block 3)  No: (blocks 5 and 6) Block 5 used in this study. |
| 45 | Emergency | Perforated diverticular disease | Yes: very scant inflammation | No |
| 46 | Emergency | Perforated diverticular disease | Yes: a number show severe acute-on-chronic inflammation | Yes: (blocks 1-4)  No: (blocks 5-8) Block 6 used in this study. |
| 47 | Emergency | Perforated diverticular disease | No | No |
| 48 | Emergency | Perforated diverticula | Yes: single focal area of neutrophils | No |
| 49 | Emergency | Perforated diverticular disease sigmoid | Yes: moderate active inflammation. Others: moderate acute-on-chronic inflammation | Yes: (blocks 3- 5)  No: (block 6)  Block 6 used in this study. |
| 50 | Emergency | Perforated diverticulitis | Yes: there is inflammation | Yes |
| 51 | Emergency | Perforated diverticulitis | Yes: active diverticulitis | Yes: (blocks 1-2) No: (block 5)  Block 5 used in this study. |
| 52 | Emergency | Perforated diverticulitis | Yes: active diverticulitis | No |
| 53 | Emergency | Perforated diverticulitis | Yes: active diverticulitis | Yes |
| 54 | Emergency | Perforated diverticulitis | No | No |
| 55 | Emergency | Perforated diverticulitis - sigmoid | Yes: but in adjacent fat and muscularis | Yes: (blocks 2-5)  No: (blocks 6-7)  Block 7 used in this study. |
| 56 | Emergency | Perforated diverticulitis | Yes: neutrophils | No |
| 57 | Emergency | Perforated diverticulitis | Yes: acute inflammation | No |
| 58 | Emergency | Perforated diverticulitis | No | No |
| 59 | Emergency | Perforated diverticulitis - sigmoid | Yes: focal active inflammation | No |
| 60 | Emergency | Perforated diverticulitis - sigmoid | Yes: inflammatory cell infiltrate on serosa | Yes |
| 61 | Emergency | Perforated diverticulitis - sigmoid | Yes: multiple foci of peridiverticular inflammation | No |
| 62 | Emergency | Perforated diverticulitis | Yes: diverticulitis | Yes: (blocks 1-3)  No: (block4-5)  Block 4 used in this study. |
| 63 | Emergency | Perforated diverticulitis | No | No |
| 64 | Emergency | Perforated diverticulitis - sigmoid | Yes: acute diverticulitis/neutrophil abscess | Yes: (blocks 5-6)  No: (block 7)  Block 5 used in this study. |
| 65 | Emergency | Perforated diverticulitis - sigmoid | No | No |
| 66 | Emergency | Diverticulitis | No | No |
| 67 | Emergency | Diverticulitis | Yes: active diverticulitis, other uncomplicated. | No |
| 68 | Emergency | Inflammatory mass - diverticular disease | Yes: active diverticulitis | No |
| 69 | Emergency | Recurrent diverticulitis | No | No |
| 70 | Emergency | Sigmoid volvulus | Yes: focal inflammation including foreign body giant cells | No |
| 71 | Emergency | Sigmoid volvulus | No | No |
| 72 | Emergency | Bowel obstruction - stricture | No | No |
| 73 | Emergency | Sigmoid stricture + perf pos colonoscopy | Yes: aggregates of suppurative inflammation within the subserosal fat | No |
| 74 | Emergency | Sigmoid stricture | Yes: one diverticulum is inflamed and foreign body giant cell reaction | No |
| 75 | Emergency | Sigmoid stricture - diverticular disease | No | No |
| 76 | Emergency | Stricture sigmoid - diverticulitis | No | No |
| 77 | Emergency | Colovesical fistula +abscess | No | No |
| 78 | Emergency | Colovesical fistula - diverticular disease | Yes: multiple diverticula actively inflamed | Yes |
| 79 | Emergency | Sigmoid perforation carcinoma + diverticulitis | Yes: acute inflammation | No |
| 80 | Emergency | ? necrotic descending colon - bowel obstruction | No | No |
| 81 | Emergency | Sigmoid carcinoma - obstruction | No | No |
| 82 | Emergency | Perforated - sigmoid carcinoma | No | No |

**Table S3. Cell count differences between control cohorts.**

| **Groups** | **Diverticula controls** | | **Non-diverticula controls** | |
| --- | --- | --- | --- | --- |
| n= | 82 | | 10 | |
| Age (mean (range)) | 68 | | 67.9 | |
| (median) | 71.5 | | 68.5 | |
| Sex (M/F ratio) | 44/41 | | 5/5 | |
| **CELL COUNT ANALYSIS** | **Diverticula controls** | **Non-diverticula controls** | | **Comparison** |
| **Eosinophil Interobserver variability** | | | | |
| \| **Table Analyzed** \| **Interobserver variability eos** \| \| --- \| --- \| \|  \|  \| \| **Column B** \| **Data Set-B** \| \| **vs.** \| **vs.** \| \| **Column A** \| **Data Set-A** \| \|  \|  \| \| **Paired t test** \|  \| \| **P value** \| **0.48** \| \| **P value summary** \| **ns** \| \| **Significantly different (P < 0.05)?** \| **No** \| \| **One- or two-tailed P value?** \| **Two-tailed** \| \| **t, df** \| **t=0.78, df=4** \| \| **Number of pairs** \| **5** \| \|  \|  \| \| **How big is the difference?** \|  \| \| **Mean of differences (B - A)** \| **0.40** \| \| **SD of differences** \| **1.1** \| \| **SEM of differences** \| **0.51** \| \| **95% confidence interval** \| **-1.0 to 1.8** \| \| **R squared (partial eta squared)** \| **0.13** \| \|  \|  \| \| **How effective was the pairing?** \|  \| \| **Correlation coefficient (r)** \| **0.99** \| \| **P value (one tailed)** \| **<0.001** \| \| **P value summary** \| ******* \| \| **Was the pairing significantly effective?** \| **Yes** \| | | | | |
| **Neutrophil Interobserver variability** | | | | |
| \| **Table Analyzed** \| **Interobserver variability neut** \| \| --- \| --- \| \|  \|  \| \| **Column B** \| **Data Set-B** \| \| **vs.** \| **vs.** \| \| **Column A** \| **Data Set-A** \| \|  \|  \| \| **Paired t test** \|  \| \| **P value** \| **0.37** \| \| **P value summary** \| **ns** \| \| **Significantly different (P < 0.05)?** \| **No** \| \| **One- or two-tailed P value?** \| **Two-tailed** \| \| **t, df** \| **t=1.0, df=4** \| \| **Number of pairs** \| **5** \| \|  \|  \| \| **How big is the difference?** \|  \| \| **Mean of differences (B - A)** \| **-0.60** \| \| **SD of differences** \| **1.3** \| \| **SEM of differences** \| **0.60** \| \| **95% confidence interval** \| **-2.3 to 1.1** \| \| **R squared (partial eta squared)** \| **0.20** \| \|  \|  \| \| **How effective was the pairing?** \|  \| \| **Correlation coefficient (r)** \| **Vertical line** \| \| **P value (one tailed)** \|  \| \| **P value summary** \|  \| \| **Was the pairing significantly effective?** \|  \| | | | | |
| **Lymphocyte interobserver variability** | | | | |
| \| **Table Analyzed** \| **Interobserver varaibility lymph** \| \| --- \| --- \| \|  \|  \| \| **Column B** \| **Data Set-B** \| \| **vs.** \| **vs.** \| \| **Column A** \| **Data Set-A** \| \|  \|  \| \| **Paired t test** \|  \| \| **P value** \| **0.84** \| \| **P value summary** \| **ns** \| \| **Significantly different (P < 0.05)?** \| **No** \| \| **One- or two-tailed P value?** \| **Two-tailed** \| \| **t, df** \| **t=0.22, df=4** \| \| **Number of pairs** \| **5** \| \|  \|  \| \| **How big is the difference?** \|  \| \| **Mean of differences (B - A)** \| **0.60** \| \| **SD of differences** \| **6.2** \| \| **SEM of differences** \| **2.8** \| \| **95% confidence interval** \| **-7.1 to 8.3** \| \| **R squared (partial eta squared)** \| **0.011** \| \|  \|  \| \| **How effective was the pairing?** \|  \| \| **Correlation coefficient (r)** \| **0.96** \| \| **P value (one tailed)** \| **0.004** \| \| **P value summary** \| ****** \| \| **Was the pairing significantly effective?** \| **Yes** \| | | | | |
| **Overall interobserver variabiliy** | | | | |
| \| **Table Analyzed** \| \| **Interobserver variability all** \| \| \| --- \| --- \| --- \| --- \| \|  \| \|  \| \| \| **Column B** \| \| **Data Set-B** \| \| \| **vs.** \| \| **vs.** \| \| \| **Column A** \| \| **Data Set-A** \| \| \|  \|  \| \| \| **Paired t test** \| \|  \| \| \| **P value** \| \| **0.88** \| \| \| **P value summary** \| \| **ns** \| \| \| **Significantly different (P < 0.05)?** \| \| **No** \| \| \| **One- or two-tailed P value?** \| \| **Two-tailed** \| \| \| **t, df** \| \| **t=0.15, df=14** \| \| \| **Number of pairs** \| \| **15** \| \| \|  \| \|  \| \| \| **How big is the difference?** \| \|  \| \| \| **Mean of differences (B - A)** \| \| **0.13** \| \| \| **SD of differences** \| \| **3.5** \| \| \| **SEM of differences** \| \| **0.90** \| \| \| **95% confidence interval** \| \| **-1.8 to 2.1** \| \| \| **R squared (partial eta squared)** \| \| **0.0016** \| \| \|  \| \|  \| \| \| **How effective was the pairing?** \| \|  \| \| \| **Correlation coefficient (r)** \| \| **0.99** \| \| \| **P value (one tailed)** \| \| **<0.001** \| \| \| **P value summary** \| \| ******* \| \| \| **Was the pairing significantly effective?** \| \| **Yes** \| \| | | | | |

**Table S4. Eosinophil counts by age range. Mean (standard deviation) and p-value. * Statistically significant (compared to normal)**

|  | **Combined** | **Elective** | **Emergency** |
| --- | --- | --- | --- |
| **20-50 yrs** n= | 11 | 4 | 7 |
| Base Mean  p-value | 83 (35)  **<0.001 ***** | 87 (43)  **0.03 **** | 80 (33)  **0.001 **** |
| Neck Mean  p-value | 44 (42)  0.05 | 33 (13)  0.57 | 51 (52)  0.09 |
| Ostia Mean  p-value | 31 (19)  0.52 | 17 (4.2)  >0.99 | 41 (19)  0.39 |
| Normal Mean | 19 (11) | 16 (5.4) | 20 (13) |
| **51-70yrs** n= | 29 | 13 | 16 |
| Base Mean  p-value | 115 (62)  **<0.001 ***** | 153 (59)  **<0.001 ***** | 84 (46)  **<0.001 ***** |
| Neck Mean  p-value | 54 (41)  **0.002 **** | 72 (53)  **0.004 **** | 41 (22)  0.09 |
| Ostia Mean  p-value | 34 (26)  0.67 | 42 (25)  0.64 | 27 (27)  0.92 |
| Normal Mean | 27 (23) | 31 (23) | 23 (22) |
| **71+ yrs** n= | 34 | 12 | 22 |
| Base Mean  p-value | 92 (50)  **<0.001 ***** | 97 (48)  **<0.001 ***** | 89 (53)  **<0.001 ***** |
| Neck Mean  p-value | 35 (23)  **0.01 *** | 35 (17)  0.45 | 35 (26)  **0.03 *** |
| Ostia Mean  p-value | 24 (22)  0.55 | 32 (32)  0.63 | 20 (14)  0.88 |
| Normal Mean | 17 (13) | 19 (16) | 16 (11) |

**Table S5. Comorbidity statistics between subgroups of elective and emergency subgroup cohorts.**

|  | **Elective n(%)** | **Emergency n(%)** | **Odds Ratio (95% CI)** | **p-value** |
| --- | --- | --- | --- | --- |
| **Comorbidities** |  |  |  |  |
| **Colon cancer** | 16(43.24) | 3(6.67) | 11(3.0-36) | **0.0001 ***** |
| **IBD** | 0(0.00) | 1(2.22) | 0.0(0.0-11) | >0.99 |
| **Other colon disease** | 2(5.41) | 5(11.11) | 0.46(0.087-2.4) | 0.45 |
| **Obesity** | 3(8.11) | 6(13.33) | 0.57(0.15-2.2) | 0.50 |
| **Diabetes** | 9(24.32) | 9(20.00) | 1.3(0.45-3.7) | 0.79 |
| **Asthma/Respiratory disease** | 7(18.92) | 6(13.33) | 1.5(0.48-5.4) | 0.55 |
| **Other atopy** | 1(2.70) | 1(2.22) | 1.2(0.063-24) | >0.99 |
| **Smoking history** | 18(48.65) | 17(37.77) | 1.6(0.63-3.6) | 0.37 |
| **Treatments** |  |  |  |  |
| **Neo/adjunct therapy** | 6(16.22) | 1(2.22) | 8.5(1.2-100) | **0.042*** |
| **PPIs** | 14(37.84) | 10(22.22) | 2.1(0.82-5.2) | 0.15 |
| **NSAIDs** | 14(37.84) | 8(17.78) | 2.8(1.0-8.1) | **0.049*** |
| **Antibiotics** | 4(10.81) | 19(42.22) | 0.17(0.057-0.55) | **0.0026*** |
